# Assignment of the antibacterial potential of Ag_2_O/ZnO nanocomposite against MDR bacteria *Proteus mirabilis* and *Salmonella typhi* isolated from bone marrow transplant patients

**DOI:** 10.1007/s42770-023-01138-4

**Published:** 2023-10-06

**Authors:** Eithar El-Mohsnawy, Abdelhamid El-Shaer, Fadia El-Gharabawy, Eslam E. El-Hawary, Abd El-Raheem Ramadan El-Shanshoury

**Affiliations:** 1 Microbial Biotechnology Unit, Botany and Microbiology Department, Faculty of Science, Kafrelsheikh University, Kafrelsheikh, 33516 Egypt; 2 Nanotechnology Unit, Physics Department, Faculty of Science, Kafrelsheikh University, Kafrelsheikh, 33516 Egypt; 3 Pediatric Hematology and Oncology Department, Faculty of Medicine, Tanta University, Tanta, 31527 Egypt; 4 Bacteriology Unit, Microbiology Section, Botany and Microbiology Department, Faculty of Science, Tanta University, Tanta, 31527 Egypt

**Keywords:** Bone marrow transplant, MDR bacteria, *Proteus mirabilis* and *Salmonella typhi*, Ag_2_O/ZnO nanocomposite

## Abstract

The rate of infectious diseases started to be one of the major mortality agents in the healthcare sector. Exposed to increased bacterial infection by antibiotic-resistant bacteria became one of the complications that occurred for bone marrow transplant patients. Nanotechnology may provide clinicians and patients with the key to overcoming multidrug-resistant bacteria. Therefore, this study was conducted to clarify the prevalence of MDR bacteria in bone marrow transplant recipients and the use of Ag_2_O/ZnO nanocomposites to treat participants of diarrhea brought on by MDR bacteria following bone marrow transplantation (BMT). Present results show that pathogenic bacteria were present in 100 of 195 stool samples from individuals who had diarrhea. Phenotypic, biochemical, and molecular analysis clarify that *Proteus mirabilis* and *Salmonella typhi* were detected in 21 and 25 samples, respectively. Successful synthesis of Ag_2_O/ZnO nanocomposites with a particle enables to inhibition of both pathogens. The maximum inhibitory impact was seen on *Salmonella typhi*. At low doses (10^−5^ g/l), it prevented the growth by 53.4%, while at higher concentrations (10^−1^ g/l), *Salmonella typhi* was inhibited by 95.5%. Regarding *Proteus mirabilis*, at (10^−5^ g/l) Ag_2_O/ZnO, it was inhabited by 78.7%, but at higher concentrations (10^−1^ g/l), it was inhibited the growth by 94.6%. Ag_2_O/ZnO nanocomposite was therefore found to be the most effective therapy for MDR-isolated bacteria and offered promise for the treatment of MDR bacterial infections that cause diarrhea.

## Introduction

Stem cell transplantation is one of the important alternatives for treating patients, as recent statistics indicate that more than one million transplantation of hematopoietic stem cells took place during the past 50 years, with an expected increase in the future [1].

Bone marrow transplantation (BMTSS) has been shown to be successful for patients who have survived the transplant, with results showing that the probability of survival after 15 years of bone marrow transplant was 80%. On the other hand, the composition of the microflora showed a high number of Proteobacteria and a low number of *Staphylococcus* compared to the uncultured controls. Lung transplant recipients (LTRs) showed less abundance of *Prevotella*, *Villanelle*, *Streptococcus*, and *Gemella* than in non-transplant patients and a higher representation of Gram-negative Proteobacteria, such as *Salmonella typhi* and *Proteus*, and Gram-positive *Staphylococcus aureus* [2]. These bacteria are resistant to many antibiotics and are commonly associated with hospital contamination that is considered an additional threat to patients [3].

Treatment of severe MDR infection in critically ill or organ transplant patients requires expert and complex clinical thinking with consideration of patients’ characteristics [4]. Extended-spectrum beta-lactam, producing methicillin resistant and carbapenem resistant, is the most common mechanisms of MDR bacteria [5]. As a result of the evolution of bacterial resistance patterns, statistics have indicated that 700,000 are due to infection caused by multidrug-resistant bacteria (MDR), which is expected to reach 10 million in 2050 [6]. As a result of the emergence of resistant bacterial types as well as infections associated with biofilms, it was necessary to find out or develop new protective agents that are characterized by effectiveness and long acting against bacteria and biofilms. The antipathogenic activity of nanoparticles and nanocomposites against several pathogens takes a lot of attention, and nanotechnology has developed many fields and led the world toward impressive scientific results [7]. Hence, the current nanomaterials have become one of the most common and rapidly emerging materials in the field of medicine. Various metal NPs have shown potent antibacterial activity in several studies [8]. This is because nanoparticles and nanocomposites have remarkable properties, such as high surface area-to-volume ratio, volume, shape, and surface activity, and they exhibit superior electrical, catalytic, and optical properties. Due to those unique properties, they have shown efficacy and sophistication compared to their counterparts on a small scale, which influences antimicrobial potency and efficacy [9]. Research on the mechanism of action of nanoparticles has demonstrated their ability to selectively inhibit through interaction with bactericidal activity and ultimately kill MDR bacteria [8]. Hence, NPs showed remarkable antimicrobial activity against Gram-negative and Gram-positive pathogens, such as *Enterococcus faecalis*, *Bacillus subtilis*, *Staphylococcus epidermidis*, multidrug-resistant *S. aureus*, and *Escherichia coli* strains, and metal NPs such as Au, Al, Pt, Cu, Zn, Ti, Ag, and Ga [10, 11]. Several metal oxide NPs such as Fe_3_O_4_, MgO, SiO_2_, CuO, TiO_2_, NiO_2_, and ZnO also exhibited inhibitory properties against several bacterial species [12]. ZnO NPs displayed vigorous antimicrobial activity by releasing Zn^2+^ ions and generating ROS, owing to their electrostatic interaction and internalization. In contrast, smaller ZnO NPs increased the interaction and abrasiveness of the bacterial cell wall [13]. Toward improving the antibacterial power of metal nanoparticles, recent reports showed that bimetallic Ag/Cu and Cu/Zn and trimetallic Cu/Cr/Ni and CuO/NiO/ZnO have been exhibited significantly improved antimicrobial performance compared with monometallic NPs [14–16]. In addition to their small size and selectivity for bacteria, metal nanoparticles are effective against pathogens listed as a priority, according to the World Health Organization (WHO). Moreover, antimicrobial studies of the nanoparticles were conducted not only in vitro but also in vivo to verify their efficacy. The WHO has declared antimicrobial resistance (AMR) as one of the biggest threats to global health [17]. Around 25,000 deaths per annum have been estimated in the European Union because of AMR [18]. The present study acts to survey the most dominant multidrug-resistant pathogens associated with bone marrow transplantation and evaluate the effect of Ag_2_O/ZnO nanocomposite as an alternative powerful antibacterial agent.

## Materials and methods

### Pathogen sample isolation

Samples were collected from September 2019 to July 2020, a period of 10 months. Stool samples from bone marrow transplant recipients who had diarrhea after undergoing bone marrow (BMT) were collected via sterile cups and aseptic techniques at Tanta University EL-Faransawy Hospital. The patients may be given medicine before the transplant to prevent an adverse reaction to the transplanted cells. The transplant took place 1 to 2 days after the completion of the chemotherapy and/or radiotherapy. Patients with various diagnoses and varied in age and sex, including females between the ages of 3 and 52 and males between the ages of 6 and 62 years, were selected for our study. Samples were cultivated on nutrient and MacConkey agar plates and incubated aerobically at 37 °C for 24 h. The suspected colonies of different shapes to the next identified isolates were picked, cultivated separately, and purified by repeated subculturing on the nutrient agar medium [19].

### Phenotype and biochemical investigations

The morphological colony characteristics of pure isolates, including shape, margin, size, and color, were assessed on nutrient agar medium. Manual work was used to perform the biochemical analysis, which included the assays of catalase [20], urease [21], citrate [22], and motility in an ornithine medium [23]. By the manufacturer’s instructions, standard biochemical tests like lysine iron agar, motility, flagella, methyl red (MR), Voges Proskauer (VP), H_2_S, gas from glucose, nitrate reduction, gas, oxidase, and pigment were also conducted [24].

### Molecular identification

Molecular characterization of isolated pathogens was performed through multiplex polymerase chain reaction (PCR) using the 16S rRNA technique. The Gene JET PCR Purification Kit was used to extract and purify the 16S rRNA sequencing areas (Thermo Scientific, Cat. No. K0701). DNA template for sample (C) 0.3 μl and PCR master mix (2×) 12.5 μl make up the PCR reaction, along with forward and reverse primers (10 pmol each) and nuclease-free water (10.7 μl). The amplification was carried out in keeping with the planned program as follows: 72 °C for 40 s, 72 °C for 4 min, 95 °C for 30 s, 60 °C for 30 s, and 72 °C for 40 s and 72 °C for 10 min. The resulting sequence was electrophoresed on an agarose gel. Sequence the PCR was performed in GATC Company by the use of ABI 3730XL DNA Sequencer at Macrogen Sequencing Services, Macrogen, Seoul, South Korea. After that, the sequence was submitted to the GenBank at the NCBI site. The BLAST program and phylogenetic analysis using the tree view program were used to assess the DNA similarities of the obtained 16SrRNA gene sequence. The obtained 16S rRNA gene sequences were submitted to the gene bank at the NCBI website. The phylogenetic analysis program was used to assess the DNA similarities of the obtained 16SrRNA gene sequence by using forward and reverse sequences, and nucleotide sequence alignments were applied for the subsequent phylogenetic analysis. The MEGAX software’s neighbor join techniques were used to build the phylogenetic tree, and a bootstrap value of 100,000 was used to determine how reliable it was [25].

### Evaluation of isolated strains against broad antibiotics

*Proteus mirabilis* and *Salmonella typhi* were examined against several antibiotic kits (Sigma Scientific services, Cairo), and the inhibition zone diameters were recorded. Purified strains were spread out on the nutrient medium, and the surface of the inoculated plates was loaded with antibiotic discs of trimethoprim/sulfamethoxazole (SXT) (25 mg), ceftazidime (CAZ) (30 mg), gentamycin (CN) (10 mg), ciprofloxacin (CIP) (10 mg), piperacillin/tazobactam (PTZ) (110 mg), colistin (CL) (10 mg), azithromycin (ATM) (10 mg), ceftriaxone (CRO) (30 mg), cefepime (FEP) (10 mg), amikacin (AK) (5 mg), amoxicillin (AM) (10 mg), and imipenem (IPM) (10 mg). The plates were then incubated for 18 h at 37 °C. Five duplicates of each antibiotic’s inhibition zones were determined and recorded.

### Fabrication and characterization of Ag_2_O/ZnO nanocomposites

All chemicals used in this synthesis were of analytical grade (Merck, 99.99% purity) and were used without further purification. ZnO nanorods were synthesized according to the protocol of El-Shaer et al. [26]. 0.4 g of polyethylene glycol and 1.8 g of silver nitrate are dissolved in 100 ml of deionized water. A mixture is stirred for 20 min followed by dropping 100 ml of 0.5 M of hydroxide sodium. The resulting ZnO/Ag_2_O nanocomposite nanoparticle powder is washed several times with ethanol, followed by drying for 2 h at 100 °C to remove water residues. XRD (Shimadzu 6000) with 2*θ* ranging from 20 to 80 °C and SEM [JSM-651OLV] were examined for detecting the crystal structures and the morphology of obtained nanocomposites.

### Evaluation of isolated strains against broad antibiotics and Ag_2_O/ZnO nanocomposites

Both pathogens were incubated individually in a nutrient broth medium containing 0.1 mg/ml, 0.01 mg/ml, 0.001 mg/ml, 0.0001 mg/ml, and 0.00001 mg/ml Ag_2_O/ZnO nanocomposites. Medium free of nanocomposites was used as a control. After inoculation, cultures were incubated at 37 °C with a shaking rate of 60 r/min for 48 h. The optical density was recorded at 600 nm using a spectrophotometer (Jasco, J-730).

### Statically analysis

All tests were carried out three times, and the means were used for statistical analysis. Utilizing Duncan’s multiple range test (DMRT) and SPSS software at a significance level of *P* < 0.05, the data were analyzed.

## Results

### The frequency and abundance of pathogens from certain patients

The patients who received bone marrow transplants and maintained diaries were included in the investigation. Sepsis was determined in patients based on microbiological testing and clinical symptoms. According to the obtained results, 100 samples out of 195 stool samples tested positive for the abundance of pathogenic bacteria, with a percentage of about 51%, whereas 95 samples out of 195 stool samples tested negative, with a percentage of about 49% (Table 1). Male children showed a high existing percentage of pathogenic bacteria (61.5%), while female children exhibited the lowest percentage (48.7%). *Salmonella typhi* and *Proteus mirabilis* were found to be the most prevalent bacterial pathogens in the sampled feces. Of a total of 195, *Salmonella typhi* and *Proteus mirabilis* were identified 25 times (13%) and 21 times (11%) of the total collected samples, respectively (Table 1).

**Table 1:** The abundance and percent of *Salmonella typhi* and *Proteus mirabilis* in bone marrow transplanting patients

Patients		Total samples	Total positive samples	*Salmonella typhi*	*Proteus mirabilis*	Other pathogens	Negative sample	% positive	% negative
Male	Child (3)	39	24	5	4	15	15	61.5%	38.5%
	Adult (7)	91	42	10	8	24	49	46.2%	53.8%
Female	Child (3)	39	19	4	5	10	20	57.7%	51.3%
	Adult (2)	26	15	6	4	5	11	57.7%	42.3%
Total		**195**	**100**	**25**	**21**	**54**	**95**	**51.3%**	**48.7%**

### Bacterial identification

#### Phenotypic and biochemical characteristics

Purified suspected *Salmonella typhi* appeared as 2–3 mm circular greyish white colonies on solid nutrient agar media and gave pink colonies (on MacConkey media), while suspected *Proteus mirabilis* appeared creamy (on nutrient solid agar), colorless colonies (on MacConkey media). The examination of the biochemical reaction of *S. typhi* exhibited positive results in the case of catalase, MR (methyl red), and nitrate reduction test (Table 2), while it had a negative reaction against citrate, oxidase, and urease test. During the examination, the biochemical reaction of *P. mirabilis* exhibited positive results in the case of the citrate, catalase, and urease test (Table 2), while it had a negative reaction against oxidase, VP (Voges Proskauer), and indole test.

**Table 2:** Biochemical analysis of *Salmonella enterica* and *Proteus mirabilis* isolated from the feces of bone marrow transplant recipients

Gram stain	Motility	Oxidase	Catalase	Indole	Methyl red	Urease	Glucose	Sucrose	Lactose	Maltose	Citrate	H_2_S	Gas	Nitrate reduction	VP (Voges Proskauer)	Pigment	Strains
-ve	-ve	-ve	+ve	-ve	+ve	-ve	+ve	-ve	-ve	-ve	-ve	+ve	-ve	+ve	-ve	-ve	*Salmonella typhi*
-ve	+ve	-ve	+ve	-ve	+ve	+ve	+ve	-ve	-ve	-ve	+ve	+ve	+ve	+ve	-ve	-ve	*Proteus mirabilis*

#### 16S rRNA sequence analysis and phylogenetic tree for molecular identification

**Fig. 1:**
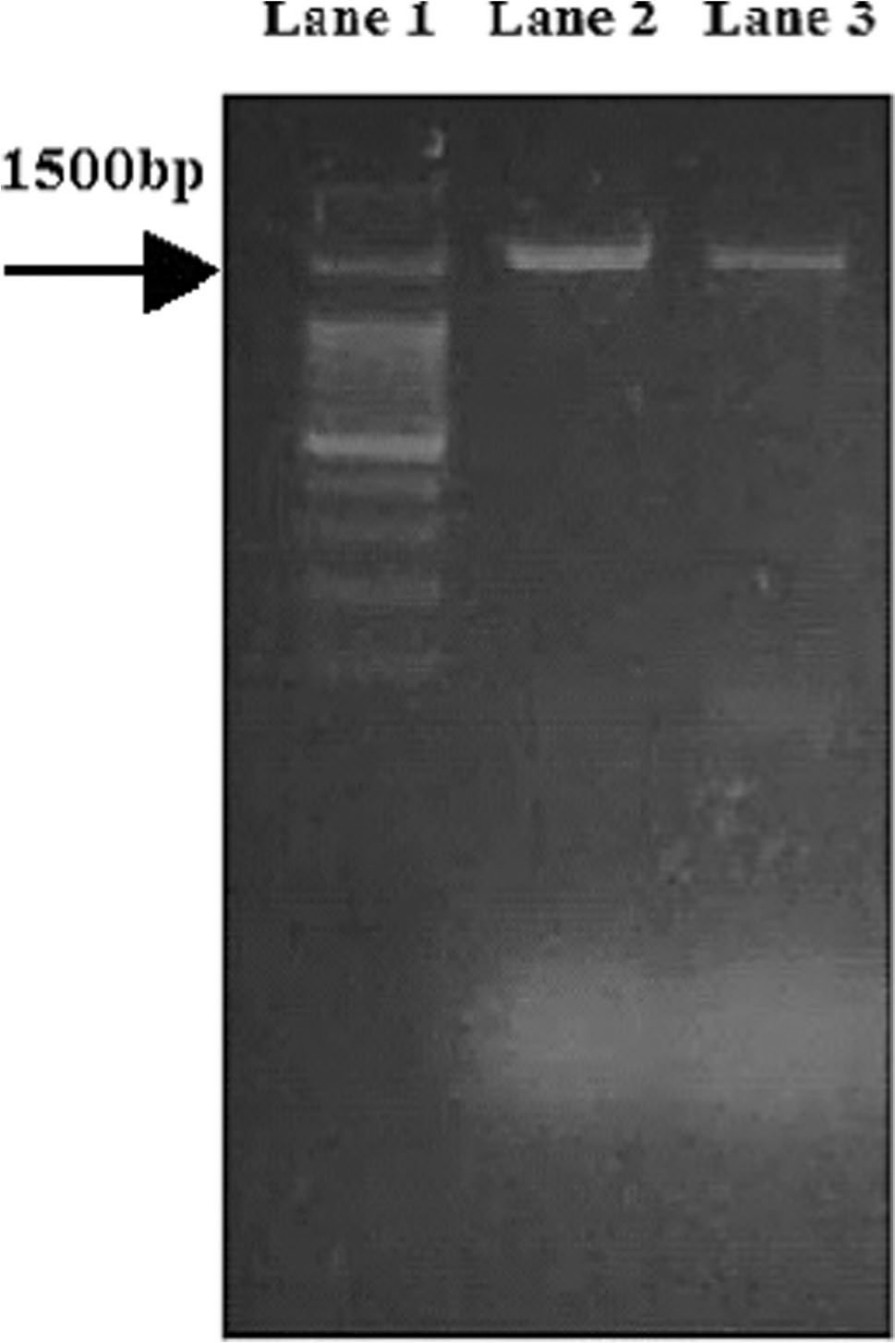
PCR amplified 16S rRNA gene. Lane 1: Sizer-1000 DNA marker, Lane 2: the amplified DNA fragment of *Salmonella typhi*, and Lane 3: the amplified fragment of *Proteus mirabilis*

**Fig. 2:**
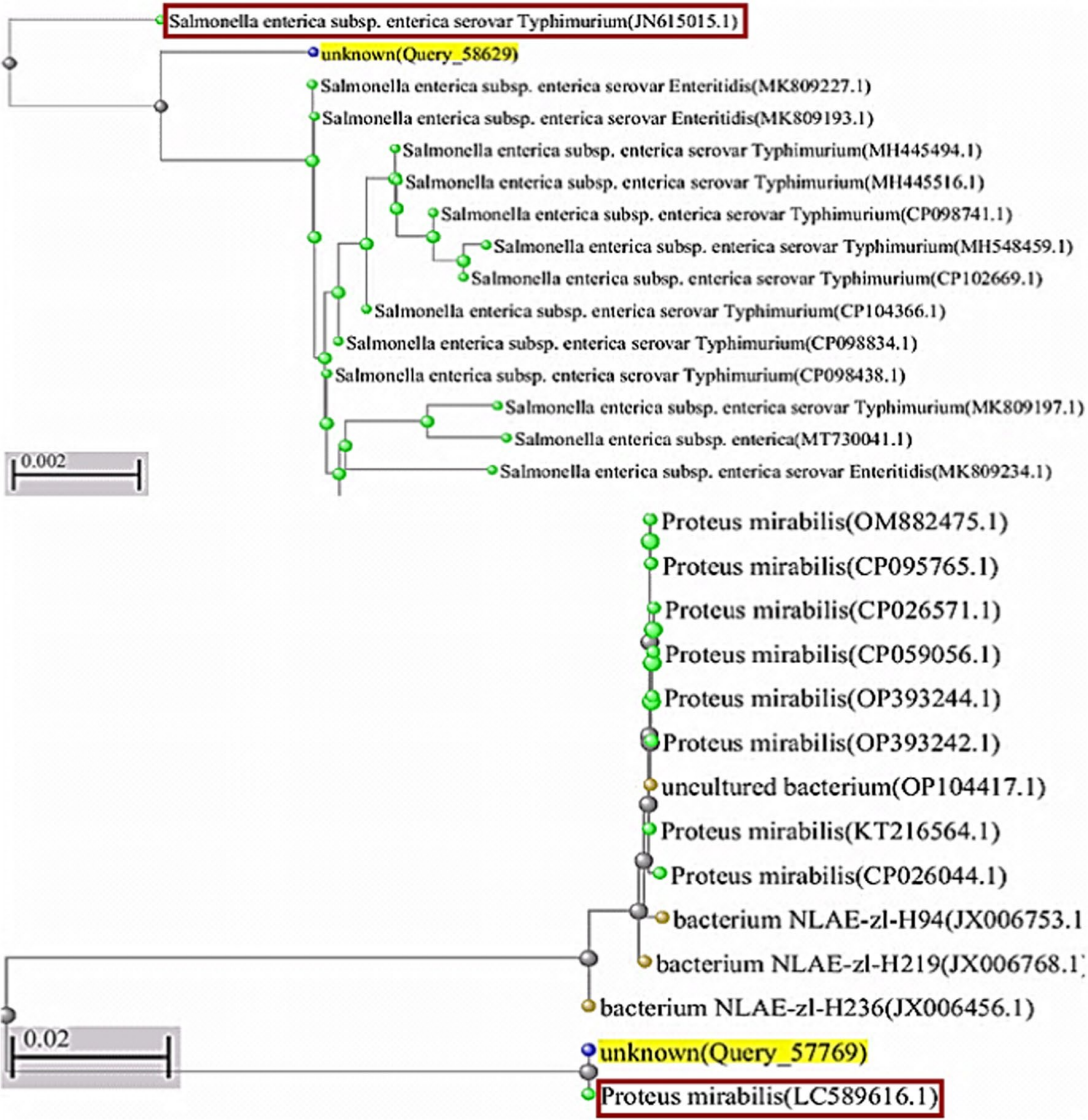
Phylogenetic tree of *Salmonella typhi* (JN_615015.1) and *Proteus mirabilis* (LC _589616.1), showing the similarity degree with other neighbor strains. Phylogenetic tree construction of the infectious bacterial isolates made in MEGA 5 software using the neighbor-joining method and an outsource group

The PCR products of the isolates suspected to be *Salmonella typhi* and *Proteus mirabilis* are shown as sharp bands at around 1500 bp (Fig. 1). For confirming the identification of examined bacteria, the 16S rRNA sequences were compared and aligned with sequences published in the NCBI Greenback database using BLAST. The 1500-bp DNA fragment was amplified, and the resultant product (Fig. 2) revealed 98% as *Salmonella typhi* (NR_615015.1), and the 1500-bp DNA fragment was amplified, and the resultant product (Fig. 2) revealed 99% as *Proteus mirabilis* (NR _589616.1). Most of the functional genes of both strains are also conserved in the reference strains with the most neighbor strains and the reference strains based on sequence similarity (Fig. 2).

#### Evaluating isolated strains for resistance to standard antibiotics

**Fig. 3:**
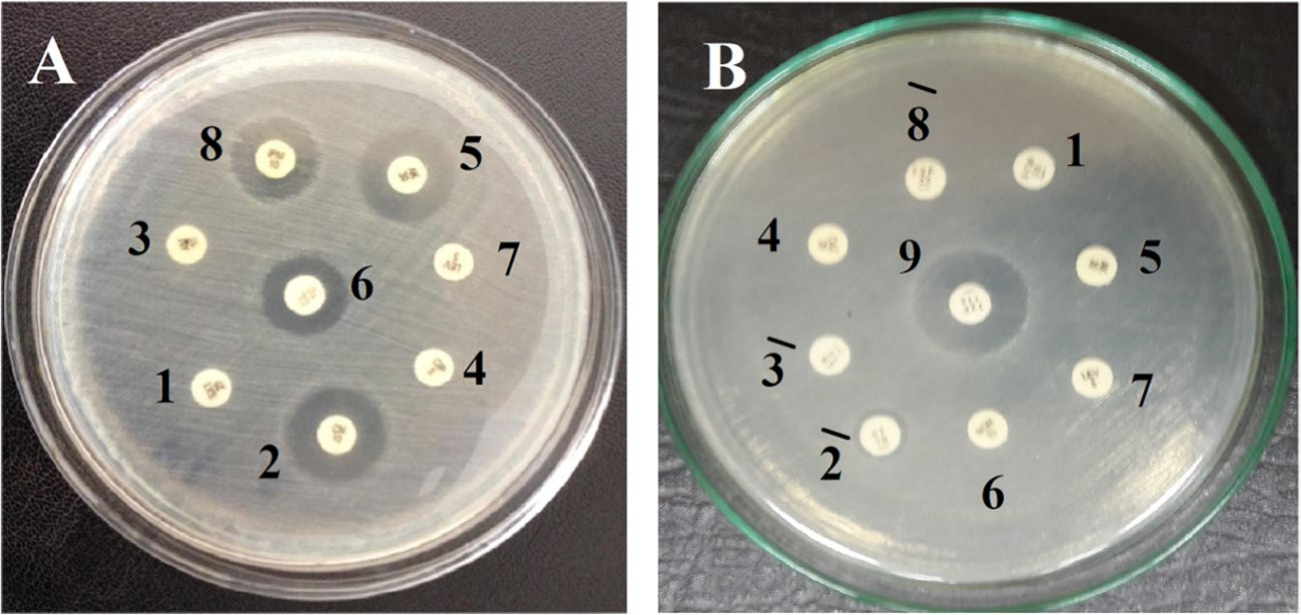
The sensitivity test of *Salmonella typhi* (**A**) and *Proteus mirabilis* (**B**) against different antibiotics. SXT (1), CAZ (2), CN (2¯), ATM (3), AM (3¯), CIP (4), AK (5), TZP (5¯), CL (6), LEV (7), IPM (8), and TZP (9)

While *Salmonella typhi* exhibited high sensitivity against amikacin (AK) (5), gentamycin (CN) (2), Cole stein (CL) (6), and imipenem (IPM) (8), it demonstrated high resistance against trimethoprim/sulfamethoxazole (SXT) (1), amoxicillin (AM) (3¯), levofloxacin (LEV) (7), and ciprofloxacin (CIP) (4) (Fig. 3A). Conversely, *Proteus mirabilis* exhibited low resistance against gentamycin (GT) (2¯) and high resistance to levofloxacin (LEV) (7), amikacin (AK) (5), ciprofloxacin (CL) (6), ceftazidime (CAZ) (3), cefepime (FEP) (8¯), trimethoprim/sulfamethoxazole (SXT) (1), and ciprofloxacin (4), while it showed high sensitive toward piperacillin/tazobactam (TZP) (9) (Fig. 3B).

### Properties of fabricated Ag_2_O-ZnO nanocomposites

**Fig. 4:**
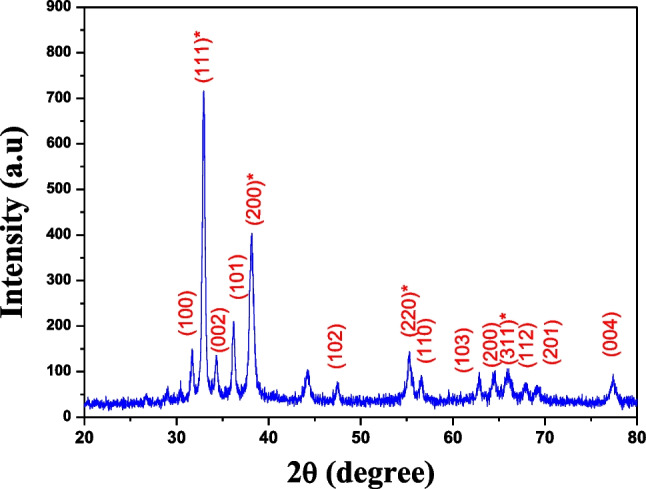
X-ray diffraction patterns of Ag_2_O-ZnO nanocomposites

**Fig. 5:**
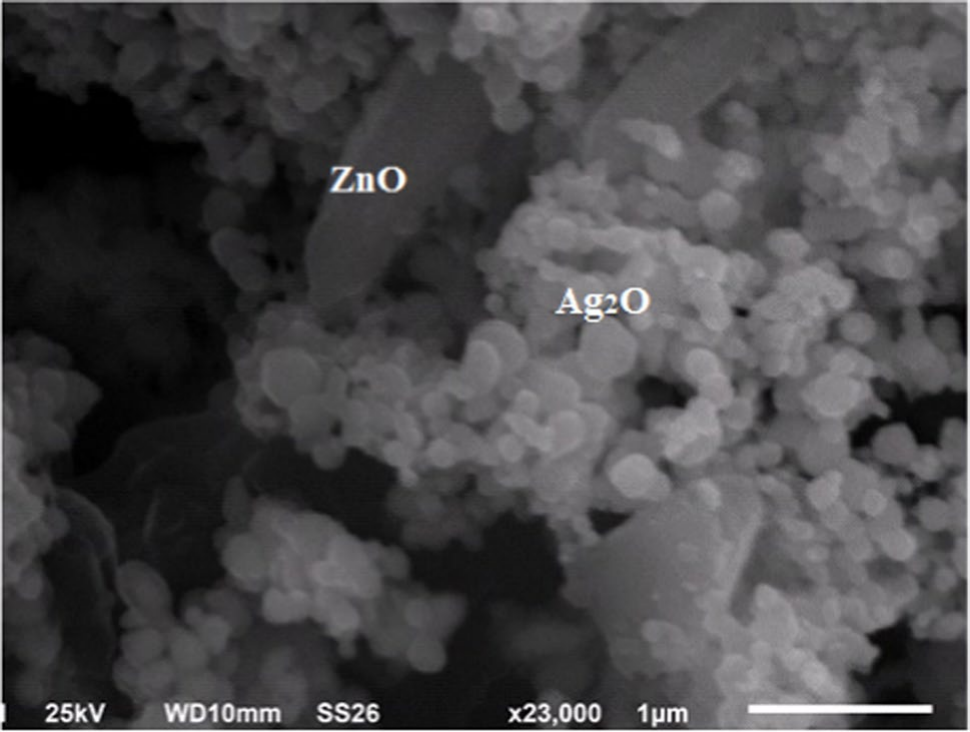
Top surface view of SEM image of Ag_2_O-ZnO nanocomposites

XRD patterns of ZnO nanorods/Ag_2_O nanocomposites shown in Fig. 4 reveal the diffraction peaks at 2*θ* values of 31.8°, 34.4°, 36.3°, 47.5°, 57°, 62.9°, 66.4°, 67.9°, 69°, and 77°, which are corresponding to the (100), (002), (101), (102), (110), (103), (200), (112), (201), and (202) lattice planes, respectively. This result designates hexagonal wurtzite ZnO crystal which is the same as reported in card no. JCPDS 36-1451. Moreover, the observed peaks of 32.99°, 38.11°, 55.24°, and 65.96° which correspond to (111), (200), (220), and (311) direction planes marked with asterisk (*), respectively, indicate the formation of face-centered cubic (FCC) structure of Ag_2_O nanoparticles which agrees very well with JCPDS card no. (76-1393). SEM image of fabricated composites showed Ag_2_O nanoparticles as spherical ball like where accumulated on the surface of hexagonal shapes of ZnO nanorods (Fig. 5).

### Controlling of MDR bacteria by Ag_2_O-ZnO nanocomposites

Identified multidrug-resistant pathogens, *Salmonella typhi*, and *Proteus mirabilis* were treated separately by Ag_2_O-ZnO nanocomposite. The growth of both pathogens exposed to different Ag_2_O-ZnO nanocomposite concentrations was monitored via optical density at 600 nm. Obtained results showed a significant inhibitory effect of all tested Ag_2_O-ZnO concentrations on both investigated pathogens in liquid media. Observed results showed the revised relation between Ag_2_O-ZnO concentrations and growth rate (Table 3). The different dilutions of Ag_2_O-ZnO composites were prepared from 10^−1^ to 10^−5^; Ag_2_O-ZnO composites were most effective against the clinically isolated. The minimum inhibitory concentration (MIC) of *S. typhi* was 10^−5^, while *P. mirabilis* MIC was 10^−4^. The activity of ZnO/Ag_2_O nanocomposites gradually decreased with the increase of dilution.

**Table 3:** Effect of different Ag_2_O/ZnO nanocomposite concentrations on the growth of *Salmonella typhi* and *Proteus mirabilis*

Ag_2_O/ZnO conc. (mg/ml)	Optical density *Salmonella typhi*	% growth of *Salmonella typhi*	Optical density *Proteus mirabilis*	% growth of *Proteus mirabilis*
Control	0.3350 ± 0.0003	100%	0.6601 ± 0.0004	100%
10^−5^	0.1563 ± 0.0002	46.66%	0.1405 ± 0.0001	21.28%
10^−4^	0.0444 ± 0.0002	13.25%	0.1230 ± 0.0003	18.63%
10^−3^	0.0332 ± 0.0004	9.91%	0.0572 ± 0.0002	8.67%
10^−2^	0.0263 ± 0.0002	7.85%	0.0448 ± 0.0004	6.79%
10^−1^	0.0151 ± 0.0003	4.51%	0.0357 ± 0.0002	5.41%

Growth was estimated as optical density at 600 nm

## Discussion

*Salmonella typhi* and *Proteus mirabilis* were very common in 195 stool samples taken from BMT patients at Tanta University Hospital, Faransavi. The main reason for high post-transplant infection may be due to immunodeficiency in post-surgery patients [27, 28]. Isolated strains were identified using morphological, biochemical, and molecular protocols of 16S rRNA genes amplified by PCR, and the NCBI GenBank revealed that the purified strains are *Salmonella typhi* (NR_615015.1) [29, 30] and *Proteus mirabilis* (NR_589616.1) [30]. The observed high resistance to multiple antibiotics exhibited by both pathogens has also been observed and confirmed by Wang et al. and Rahdar et al. who suggested some defenses, including restricted permeability and egress of b-lactams, quinolones, and aminoglycosides, which are the main characteristics of these pathogens [31, 32]. In addition, there are spontaneous mutations that lead to increased expression of chromosomal β-lactamase genes. The growing recognition of the role of efflux systems in overall antibiotic resistance has led to the search for efflux pump inhibitors as therapeutic agents. In addition, another control strategy is the formation of biofilm communities; the successful preparation of the Ag_2_O/ZnO nanocomposite was attributed to the wurtzite hexa Ag_2_Onal ZnO crystal, which is identical to the crystal listed in card no. JCPDS 36-1451 [33, 34]. Also, the observed peaks at 32.99°, 38.11°, 55.24°, and 65.96°, corresponding to the orientation planes (111), (200), (220), and (311), respectively, prove the formation of a FCC which emphasize that Ag_2_O nanoparticles come in agreement with the JCPDS card number (76-1393) [34]. The effective antibacterial power of Ag_2_O/ZnO nanocomposites is due to Ag_2_O and ZnO. Since zinc has shown high antimicrobial properties [35], it is widely used as sunscreen, skin ointments, anti-dandruff shampoos, etc. [36]. The US Food and Drug Administration has recognized the safety of zinc oxide as an antimicrobial agent (FDA, 2011). In contrast, silver oxide is another strong antimicrobial compound, and the inhibitory capacity is increased by reducing the particle size [37, 38]. Therefore, it is believed that silver oxide and zinc oxide may have a synergistic effect in the form of nanocomposites. The inhibition mechanisms involve direct contact of NPs with cell walls and subsequent destruction of the bacterial cell wall; release of antimicrobial ions, mainly Zn^2+^ and Ag^+^ ions; and ROS mobility [39, 40]. However, the toxicity process of the ZnO·Ag^2^O nanocomposite may be due to the negative charge of the bacterial cell wall, which promotes the absorption of the positive charges of silver and zinc ions. This leads to electrostatic interactions, which consequently form ion leakage into the microbial cell wall and its destruction [41, 42]. On the other hand, ZnO·Ag_2_O nanocomposites can interfere with enzyme production by generating reactive oxygen species (ROS) that directly affect DNA transcription and translation [43].

## Conclusion

The current study found that 100 pathogens were isolated from 195 stool samples from diarrhea patients, indicating that infection rates for bone marrow transplant patients are high. Multidrug-resistant bacteria, *Proteus mirabilis* along with *Salmonella typhi*, were found in 21 and 25 samples, respectively, based on morphological, biochemical, and molecular analysis of the isolated pathogens. The Ag2O/ZnO nanocomposite material was found to be an effective inhibitor, with *Salmonella typhi* inhibited by 53.4% in 10–5 g/l and 95.5% in 10–1 g/l. *Proteus mirabilis*, on the other hand, was inhibited by 78.7% at 10–5 g/l and 94.6% at 10–1 g/l.
